# Floor Covering and Surface Identification for Assistive Mobile Robotic Real-Time Room Localization Application

**DOI:** 10.3390/s131217501

**Published:** 2013-12-17

**Authors:** Michael Gillham, Gareth Howells, Sarah Spurgeon, Ben McElroy

**Affiliations:** Engineering and Digital Arts, University of Kent, Canterbury CT2 7NT, UK; E-Mails: W.G.J.Howells@kent.ac.uk (G.H.); S.K.Spurgeon@kent.ac.uk (S.S.); bm208@kent.ac.uk (B.M.)

**Keywords:** mobile robotics, floor features, optical mouse, room localization

## Abstract

Assistive robotic applications require systems capable of interaction in the human world, a workspace which is highly dynamic and not always predictable. Mobile assistive devices face the additional and complex problem of when and if intervention should occur; therefore before any trajectory assistance is given, the robotic device must know where it is in real-time, without unnecessary disruption or delay to the user requirements. In this paper, we demonstrate a novel robust method for determining room identification from floor features in a real-time computational frame for autonomous and assistive robotics in the human environment. We utilize two inexpensive sensors: an optical mouse sensor for straightforward and rapid, texture or pattern sampling, and a four color photodiode light sensor for fast color determination. We show how data relating floor texture and color obtained from typical dynamic human environments, using these two sensors, compares favorably with data obtained from a standard webcam. We show that suitable data can be extracted from these two sensors at a rate 16 times faster than a standard webcam, and that these data are in a form which can be rapidly processed using readily available classification techniques, suitable for real-time system application. We achieved a 95% correct classification accuracy identifying 133 rooms' flooring from 35 classes, suitable for fast coarse global room localization application, boundary crossing detection, and additionally some degree of surface type identification.

## 1. Introduction

Autonomous robotic systems function well in a carefully defined workspace. However, assistive devices such as robotic wheelchairs need to consider user requirements whilst negotiating highly dynamic and varied arenas, particularly as indoor activity is highly room correlated. Thus, for any effective assistive system a robust degree of real-time localization becomes essential. Obtaining and maintaining online coarse self-localization would allow assistive systems to select appropriate navigation strategies such as when approaching doorways and waypoints or following corridors, and to know precisely when room boundaries are crossed; more importantly maintaining coarse localization allows the system and human to converse using the exact same terms and to communicate that information to other automated systems or human assistants. Localization can be achieved using Global Positioning Satellites (GPS) or mobile telephony techniques. However, the degree of accuracy and loss of signal can present a real problem within buildings, particularly when there is a need to differentiate between small rooms as is common in domestic situations. Tracking and localization within a room has been covered extensively within the literature [1,2]. While current research favors optical methods [3], Wi-Fi systems are however widely employed and considered by many a *de facto* standard method [4]. Mobile robotic localization research for systems employing limited short range sensors is lacking in the literature [1]. Any robotic application must have an executable trajectory, and autonomous robotic devices require reference points and maps for localization and navigation, whether those data are known *a priori* or obtained dynamically whilst undertaking exploration. However assistive technologies such as electric wheelchairs are drawing mobile robotic interactions increasingly towards the uncertain and complex human environment. Seamless crossover between human defined-desired trajectories and autonomous system aided trajectories is required, human assistive systems have the intelligent user in the loop [5,6] which necessitates abandoning fixed definable workspaces—best suited to autonomous robotics—and instead adopting stochastic and semantic based workspaces [7]. Methods commonly employed in the Euclidean geometric domain, such as covariance ellipses indicating location and object uncertainty, now for assistive technologies require weighted nuances; obstacles and targets thus having a spectrum of importance. Whilst Cartesian maps provide a useful reference, and must be accurate, allowing interaction with fixed infrastructure, localized dynamic interactions within the human environment are perceptual, subjective and instinctive and therefore any robotic assistive system must incorporate some form of learned localized perceptive temporal mapping in order to be effective. When the assistive device is first initialized, for example after powering down and then having been manually moved, localization becomes the first dictate; current methods require some form of scanning or initial exploration to generate a map which is then compared with a stored map. However this approach requires some time and unnecessary motion, both undesirable features in any human assistive system. In addition a habitable room may be cluttered and dynamically varying hence geometric mapping will not remain consistent over time.

In this paper we present a novel and real-time method of room recognition based upon the flooring color and texture. Rigorous testing has been undertaken to establish whether floor feature consistency is sufficiently robust in typical human environments. The method is tested and evaluated on challenging data sets acquired in real home, office and public dynamic environments.

## 2. State-of-the-Art

Whilst much work has been done in the field of robot self-localization, significant difficulties remain with integration into the dynamic human world. Techniques such as Radio Frequency Identification (RFID) tags [8] and Wireless Fidelity (Wi-Fi) [9] have been introduced in the healthcare field to monitor patient and staff locations. Rimminen *et al.* [8] used capacitive RFID tags embedded in the shoes of nurses and an electric field floor sensor; they reported 93% successful localization. Doshi-Velez *et al.* [9] mounted devices on wheelchairs aiming to reduce the time spent locating patients in a residential home; they reported significant time savings, but they also indicated 8.9% false positives where the radio signal was not being bounded by walls. Jiang *et al.* [10] developed an occupancy clustering technique utilizing Wi-Fi signatures for room distinguishability; they reported 95% successful location identification.

Most locations frequented by wheelchair users, such as their homes or those of friends, offices, and other public places, are unlikely to have such infrastructure and even if domestic Wi-Fi is utilized, there is a possibility of it being turned off, obstructed, or moved. Thus a more robust room identification solution, less reliant on specialized infrastructure, must be sought for any practical mobile robotics system particularly if it is to be effective in diverse and dynamic environments.

Ceiling lights and tiles [11–13] have all been used in the literature to provide a means of localization within a room. However, lighting conditions can prove problematic and not all rooms have multiple lights and suspended ceilings. Other localization techniques have involved sonar mapping [14]; these require room scanning, thus inducing unwanted motion and delay before identification is possible, as do laser range finding LIDAR methods. A well-established camera-based image feature matching method, Speeded-Up Robust Features (SURF) [15] employed by Murillo *et al.* [16], was used to localize a robot. The method compared the current omnidirectional image with stored images and they reportedly achieved a 95% robot tour room recognition rate.

Any assistive or autonomous robotic system requires localization information prior to action; path planning can only be achieved from knowing the current location relative to other locations, and is thus an essential component for any trajectory generation or assistance. Localization and tracking is often carried out through GPS and/or GSM, or other radio beacon systems. However loss of signal often occurs in buildings, and when available is usually limited to an oval probability footprint several meters by several meters, with little regard to room walls and boundaries. Therefore any radio based system gives rise to false positives, and false negatives, when considering a specific room; thus any localization system solely utilizing these methods suffers susceptibility to false reporting, other methods of localization not involving radio systems require exploration time or delicate expensive rotating sensors and are thus unsuitable for human assistive devices; image processing localization techniques are computationally expensive and have restrictive coverage. Therefore determining which room, for example in which house or apartment in a multistory terrace or block, in real-time to an acceptably robust degree, in a highly dynamic environment, appears difficult if not impossible to achieve.

## 3. Floor Feature Determination

Flooring is usually laid for some considerable time without change, tends to be homogeneous in color and texture patterning and has variance upon location, particularly room to room in the home or living environment and is usually kept clean and free of obstacles and clutter. Whilst hospitals and public buildings may well have the same type of flooring throughout zones, there are usually some differences, in particular color coded strips run along the corridors of many hospitals to allow people to traverse from place to place, other infrastructure may also be present or cost effectively implemented. Offices, houses, flats, shops and restaurants are where people spend the majority of their time, all of which would in all likelihood not have the infrastructure necessary for robotic localization; therefore flooring offers an additional tool in the human assistive robotic localization arsenal.

Flooring can be smooth, as in the case of a hard surface such as linoleum or wood, or rough as in carpet, providing a degree of texture, and patterning is also an important discriminator. Whilst it is entirely possible for a floor to be part covered by a rug or have a stain, these tend to be permanent features and the variance of these features could be said to be slowly changing over time, as wear and tear occurs for example; however any system reporting falsely could be easily retrained for that room, an occurrence in all likelihood equal to one introducing new locations and deleting old. Furthermore thresholds of rooms or doorways usually have carpet or flooring dividers thus further bounding the location.

Fast reliable classification requires extracting suitable robust flooring features. Previous work classifying and cataloging images in large datasets has been achieved by simply defining a red, green and blue (RGB) ratio in color space [17], thus effectively reducing an image to three single color values and standard deviation. Most flooring is much less detailed and varying as human interest photographic images are, and therefore these features are highly suited to this application. Various statistical and structural methods defining texture have been reviewed [18,19] however a true texture definition remains undefined, metrics of texture could be described as homogeneity, contrast, correlation and energy which can be obtained from a greyscale image. Therefore we have chosen to use overall image reflectivity, contrast and homogeneity as a metric of flooring texture.

## 4. Hardware

There are numerous techniques for localization utilizing a range of hardware devices, for example vision-based pattern recognition systems typically use low resolution webcams to obtain images which are then processed and parsed, with long computational time due to need for large numbers of comparative stored images, even with modern techniques [15], or lowering frame rates to improve accuracy, therefore hardware dependent problems exist for any real-time human assistive system [2]:

- Wi-Fi and radio broadcast (personal and local area networks) offer only coarse localization, and are sensitive to interference, dynamic changes and propagation effects, and require power level mapping, and suffers from a difficulty to bound rooms effectively.
- RFID is limited by range and accuracy, requires installation therefore limited to identifying rooms with the devices installed.
- Photonic devices are sensitive to ambient conditions, reflection, and obstruction and may require some infrastructure, which are all problematic for a dynamic human environment.
- Image processing has high processing requirements, sensitive to ambient lighting conditions, and environmental changes, and obstructions, although good for room identification.
- Geometrics, such as sonar, laser and infrared ranging require some degree of scanning or platform motion and all can be affected by dynamic conditions, cluttering, reflectance and scattering, although good for room identification some considerable time may elapse before identification is completed and thus not directly suitable for assistive robotic application.
- Inertial and mechanical sensors suffer from drift due to: integration, noise, thermal differences, and alignment errors, hence they require periodic calibration; they also require accurate initialization because any error in initial position is carried forward. Therefore these types of sensors are not suitable for initial system localization but would work well with a system which periodically accurately determines some position, such as a room or floor covering boundary.
- Geomagnetic sensors are strongly affected by electromagnetic fields and metallic objects and therefore highly un-reliable indoors.

**Figure 1.**
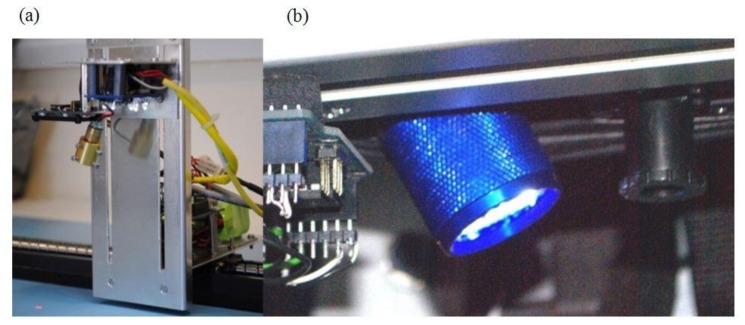
(**a**) Mouse camera with laser illumination and color sensor with white LED illumination; (**b**) Improved and final configuration with mouse camera and lens, and color sensor both illuminated by white LEDs.

Therefore for any real-time human assistive system computationally fast sampling hardware with easy to extract data must be employed for that system to succeed, and for this reason we have chosen two high speed low cost sensors to extract the RGB color features and surface texture features. For our three feature color sensor we have chosen an Avago ADJD-S311-CR999 [20] four color channel (RGB and White) surface mount photodiode array sensor comprising of filters and front end analogue-to-digital converter with adjustable integrators and offsets, connected by serial interface to a small ATmega328 microcontroller programmed to read register information from the sensors and send those values to a laptop which we used to collect those spectral data. Illumination was provided by eight white LEDs mounted around and parallel to the sensor shown in Figure 1.

Whilst our color sensor provides the spectral image processing element, we require a real-time monochrome texture sensor. Optical computer mice sensors are based upon compact high speed monochrome image processing, therefore we have chosen for our texture feature sensor an Avago ADNS-2610 1,500 fps compatible [21] MCS-12086 simple small form factor 19 × 19 pixel array optical mouse sensor. This sensor conventionally uses the optical flow algorithm [22] to return X and Y relative motion. The process of obtaining the relative motion requires hundreds of vector calculations, based upon comparing a moving pattern of each bright pixel's relationship to eight neighboring pixels between two frames. These velocity vectors are fused over a number of frames to provide a low noise resultant velocity vector, which is available in component form from the device registers. Pixel integration time is carefully managed in order to preserve the feature patterns between frames, for example previous work has shown that the whole optical mouse image can be utilized, rather than many 3 × 3 matrices, as a feature pattern [23], in order to improve performance of the motion detection algorithm. Therefore the optical mouse provides a robust and stable image pattern of the surface over which it travels.

The data registers on the sensor provide datum information each clock cycle as shown in Table 1. This particular model only makes one image pixel magnitude of information available per frame, thus 361 cycles or frames are required per accessible image. Each frame taken by the optical mouse is directly representative of a form of surface texture, where scattering of the light from an illumination source is dependent upon the surface irregularities [24] and angle of incidence.

The optical mouse sensor has a pin-hole lens restricting the focal point, and the number of photons entering the device so that the mouse only functions when in very close proximity to a surface. We initially used a laser for illumination in order to increase the distance from the surface that the optical mouse sensor was effective, Figure 1a. However, for safety reasons and practical application it was later decided to modify the optical mouse sensor and utilize the illumination from the color sensor thus effectively creating a single sensor package. The original optical mouse sensor pinhole cover was replaced by a small webcam lens to give a wider field of view whilst also allowing more light into the sensor shown in Figure 1b. Both laser and white light illumination proved equally successful as we show later. A standard 180 × 320 color webcam was chosen to provide a comparison benchmark sensor when imaging the flooring materials.

## 5. Methodology

Classification is used in statistics and machine learning systems as a method of determining to which category some current observation (the testing data set) belongs when compared with some stored or learned observation (the training data set). The Nearest Neighbor (KNN-1) classifier is well understood and used extensively due to simplicity of operation [25]. KNN-1 functions well across different ranges and types of datasets; therefore it provides a good benchmark to test flooring features. However, in practice, when processing large datasets, for example too many different types of floor, statistical classifiers often perform better; therefore the linear classifier Bayes-1, and quadratic classifier Bayes-2, and the simplistic Naïve Bayes Classifier have also been used when testing feature sets. According to research [25] all these classifiers look promising for application in any real-time pattern recognition systems, particularly the statistical classifiers.

We were able to sample the optical mouse camera and RGB sensor at a rate between 1 and 500 samples per second using the ATmega328 microcontroller, depending on the optical mouse sensor model, and microprocessor, 800 to 1,500 frames per second can be achieved [21], the color sensor can be sampled at 10,000 times per second [20] although for the purpose of these tests we recorded either single samples or when in motion approximately 50 samples every second.

The mouse camera and color sensor were mounted beneath the robotic platform together with the web camera and connected to a laptop to record the data. Calibration for the system was undertaken by using a variety of materials, dark to light in order to determine the optimum illumination level requirement, color cards were used for the RGB color. The web camera was positioned so as to center in the captured image the same area that the mouse sensor and color sensor observed.

## 6. Feature Extraction

The benchmark web camera was used simultaneously with the proposed system to image flooring samples. The collected web camera images had simplistic RGB channel color median bin values extracted as the color features. Texture was obtained from a grayscale mapping of the same color image used to extract RGB features, each greyscale image was analyzed as a gray-level co-occurrence matrix (GLCM), which is a statistical method that considers the relative spatial pixel relationships. The result of the statistical analysis provides four image texture features; a relative degree of contrast, correlation, homogeneity, and energy.

**Figure 2.**
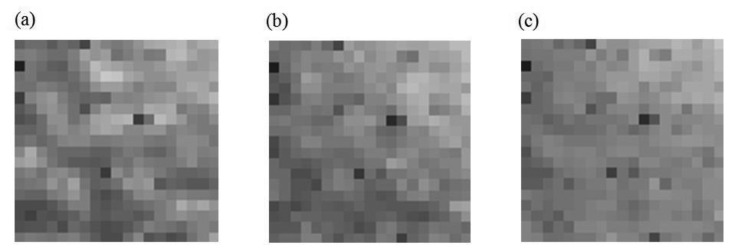
(**a**) Coarse sandpaper mouse sensor image; (**b**) Medium sandpaper mouse sensor image; (**c**) Fine sandpaper mouse sensor image. All images were obtained, using a 30 degree angled white LED illumination source, from the re-lensed mouse sensor.

The color sensor provides a single pixel for each of the RGB channels, thus comparable with the web camera RGB median binning method [26]. The complete optical mouse sensor image can be obtained from the sensor registers as a grayscale bitmap, Figure 2 shows 3 19 × 19 pixel images, from which we can also extract a GLCM and thus directly compare with the web camera. Additionally the optical mouse sensor also provides raw data which are directly representative of the surface irregularities due to the intrinsic properties needed to determine an overall velocity vector output.

One of the mouse sensor registers gives a value for surface quality (SQUAL), the value represents the total number of features identified; these features are essentially obtained by utilizing a 3 × 3 pixel mask across the whole image, pixel by pixel, excluding the edge rows and columns. An overall brightness gradient may then be determined between the central pixels of each masked matrix to its neighboring pixels within the mask; this gradient is then represented as a vector subsequently assigned to each central pixel. These feature vectors are then used by the optical flow algorithm to determine an overall motion vector of the sensor in Cartesian form between frames. Therefore the brightness contrast between pixels needs to be significant in order to be track-able as a surface feature. However in the case of the modified mouse, where each pixel images a larger surface area than the standard mouse sensor, due to the lens change, rather than image the microscopic nature of surfaces, we now image slightly more macroscopic. Figure 2 shows three different, same color, textured materials taken using the modified mouse sensor with a 30 degree illumination angle, the contrast or gradient between neighboring pixels, and overall contrast, the angled illumination slightly exaggerates the surface profile which allows surfaces with homogeneous color, hence un-patterned, to become discernible by a measure of surface roughness.

## 7. Results and Discussion

**Figure 3.**
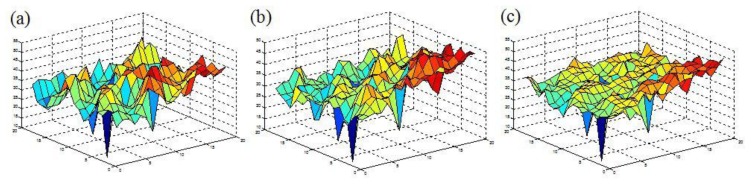
(**a**) Pixel 3D mapping of coarse sandpaper image; (**b**) Pixel 3D mapping of medium sandpaper image; (**c**) Pixel 3D mapping of fine sandpaper image.

A very simple measure of the surface texture can therefore be extracted from the mouse sensor registers, contrast, and relative brightness, can be obtained from the average, maximum, and minimum pixel values, also available from the registers each clock cycle, given in Table 1, particularly as the optical mouse sensor is intrinsically designed to maintain these relative magnitudes, by modulating the shutter period in order to keep the features consistent between frames. Surface roughness is clearly discernible in Figure 2, different grades of the same color sandpaper, coarse in Figure 2a, medium in Figure 2b and fine in Figure 2c. This surface roughness, or equally colored patterning, shows correlation with the gradient between a pixel and its neighbors, this variation can be better quantified by the 3D mappings of the three sandpaper images, shown in Figure 3, hence there is a direct relationship between the SQUAL count and the surface homogeneity.

A series of 52 different flooring coverings were obtained for an initial testing, including tightly woven carpet through to long pile, various linoleum patterns and wooden flooring. Classes were manually selected to test the ability of the sensor to correctly identify individual flooring. A 60% training dataset and 40% testing dataset were obtained by a random splitting of the collected data samples, and testing, using PR Tools 4 [27] for pattern recognition. A series of five complete runs, including random data splitting, were performed for each class and the average results tabulated in Table 2. Samples were taken uniformly across the surface of the initial flooring test using a motorized preprogrammed X/Y table with background consistent fluorescent lighting. These measures were undertaken to ensure repeatability. Tests were re-run to confirm this. The test sensor configuration is shown in; Figure 1a, a red laser is used for mouse camera surface illumination and white LEDs for the color sensor surface illumination.

The results in Table 2 for the initial floor covering test, using the un-modified mouse sensor and red laser illumination, show that the mouse sensor registers values provide an identifying fingerprint for different flooring materials, the Bayes-Normal-1 classifier giving a 38.7% correct identification, the RGB color sensor RGB features using the same classifier gave a 84.7% correct classification, and when those features are combined into one feature set a small improvement in correct classification occurs. These results were taken at a height from the surface of 70 mm, which was equally comparable to other testing previously run at surface level [28].

The initial test was repeated, six months later, using as many of the original materials as possible and with the modified mouse sensor. This testing was static, unlike the previous dynamic test, to compare our sensor results with those of a webcam and also directly the complete mouse sensor image.

The results shown in Table 2 suggest that the modified mouse sensor with white LED illumination performance is comparable and equivalent to that of the un-modified laser illuminated mouse sensor. The modified mouse sensor registers and color sensor combined give a 95% correct identification flooring classification using the Bayes-1 classifier, which compares with the previous unmodified mouse camera 87% correct Bayes-1 classification. The webcam image GLCM texture analysis and webcam color RGB binning benchmark methods gave a correct identification Bayes-1 classifier result of 85%.

**Figure 4.**
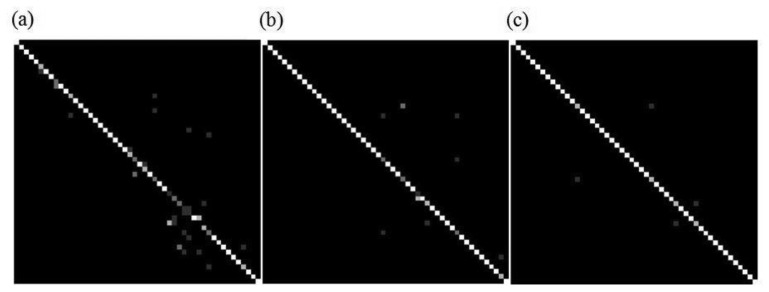
(**a**) LDC webcam color and texture confusion bitmap; (**b**) LDC RGB color sensor and mouse registers confusion bitmap; (**c**) LDC RGBW color sensor and mouse registers confusion bitmap. The vertical axes denote the classifier determined label and the horizontal axes denote the true label.

Figure 4a shows the confusion bitmap for the webcam RGB and texture combined features, Figure 4b shows the mouse sensor registers and color sensor RGB confusion matrix bitmap, and Figure 4c shows the improvement in clarity between classes, not tabulated in the results, when the fourth color channel white feature is added, suggesting further improvement is possible.

The results clearly show that flooring material identification is consistently improved when color features are combined with texture features, in some cases significantly. However the mouse camera complete image proved to be a poor method for texture determination using the GLCM method. The inability to capture all pixels on one clock cycle simultaneously is thought to be one reason. The material illuminating source spectrum significantly affects the RGB reflected values, as does directionality; therefore testing was carried out under various exaggerated lighting conditions, so that the polluting light totally overwhelmed the localized illumination source, as if there were no shading by the robotic platform. Taking 500 samples, 10 per class, it was found that even when combining sampling taken across different lighting conditions, shown in Table 3, the ability to uniquely identify four grades of similar color sandpaper and seven shades of color paper remained at 85.2% for the mouse camera and color sensor method, and 82% for the comparative webcam test. The mouse camera, whose spectral sensitivity sits closely around the 600 nm peak [21], was more tolerant of the wavelength shift occurring with the fluorescent lighting test than the color sensor, the features obtained from the mouse camera significantly improved the overall correct Bayes-1 classification result 19.2%. There are methods, not considered here, for the removal of unwanted lighting; one such mitigating method, utilizing five different room layouts, reported a 75% correct room image identification when three different lighting conditions were employed [29].

A robotic platform with the sensors mounted beneath; hence partly shaded, was utilized to take extensive sampling, with no background lighting control, across 133 rooms where the flooring was in various conditions of wear. Flooring related to a university campus accommodation and other sites, which used the exact same flooring across many rooms/locations, giving a total of 35 classes of flooring in different states of wear, and varying levels and wavelengths of background illumination were available for testing, 59,797 samples were taken across all the rooms from two directions wall to wall. The results in Table 2 show the Bayes-1 classifier identifies correctly all classes of flooring using the color sensor RGB features 76.9%, and the mouse camera register features 42.7%, and when combined improved correct identification to 85.2%. The 1-NN classifier combined features correct room identification performed even better at 94.7%. Similar work identifying rooms using images reported; home 1 with three classes correctly identified 85% and home 2 with five classes correctly identified 73% [30]. Other work 96% correctly identified six classes of floor surface materials, one example of each, 25 samples in an 80:20 training testing split, using images in HSV color space with a random tree classifier [31].

We tested the mouse sensor and color sensor with the platform in motion at various speeds from stationery up to 1m/s with no surface identification performance difference. During the extensive room flooring testing it was found that the various states of wear of identical flooring generated a unique signature, sufficient to significantly differentiate 18 rooms with originally identical flooring 58.4% correctly using the Bayes-Normal-2 classifier. We finalized our testing by running a real-time classifier on a laptop mounted on the wheelchair; we were able to detect the moment flooring boundaries were crossed, and when the joins between similar flooring was crossed.

## 8. Conclusions

The simple features and sensors we propose for flooring, and thus room identification, enables rapid processing techniques to be used. We have utilized standard pattern recognition techniques in our experimentation which according to other research, when using modest feature sets, can be used for real-time system application [25], which is highly desirable for human assistive robotic devices.

We have shown that room identification through flooring comparison to a high degree of accuracy is possible [2], although the final degree of accuracy is dependent upon the classifier chosen; according to the confusion matrices miss-identifications were from similar classes or where flooring was too low in reflectivity. Other localization methods such as those utilizing radio waves or GPS often fail to correctly define the boundaries of the room, and may give a false reading for some considerable range outside of the room [9], our method detects the moment flooring boundaries are crossed, an important requirement for correcting inertial sensor odometry drift, and our sensor arrangement covers less than 1 cm^2^ of floor at any one time, therefore small changes in platform position present entirely new floor area for sampling rather than, as with other methods, a need for significant movement around the room.

We have also shown that from the optical mouse camera extractable data: surface quality, maximum pixel, minimum pixel, and average pixel values, can be successfully used as a simple form of surface texture identification, sufficient on its own as a surface identifier, across classifiers and lighting conditions a 19%–52% correct classification rate was obtained. Although not specifically determined we suggest that further work could be done with the mouse sensor, having identified the surface from previous known samples, to use this information as feedback for wheel encoder odometry error mitigation and traction control.

When we combined the mouse sensor with the four pixel color sensor using RGB color space it was demonstrated that the combination of features improved the overall correct surface identification when compared with using the individual sensors alone. We were also able to demonstrate that when lighting conditions vary, providing flooring training samples are updated, that surface determination is maintained. This would be important in any user-in-the-loop system, when changes occur, such as new lighting or new floor stain; the user would be able to re-train the system by correcting system errors which they can easily relate to, thus creating a semantic symbiosis between user and robotic assistant.

Our experimental results demonstrate that two inexpensive sensors, with low computational requirements using simple pattern recognition techniques, can successfully distinguish different flooring materials when compared with results obtained using a conventional web camera. We therefore conclude that the two sensors are complementary with each other and can be combined for the desirable purpose of robust low cost coarse robotic localization, real-time mobile robotic surface determination, and boundary crossing applications. We also conclude that flooring offers an effective alternative room identification method, even when identical flooring was tested the level of wear and staining proved to be a unique fingerprint.

## Figures and Tables

**Table 1.** Optical mouse sensor data registers.

**Register**	**Address**	**Range**	**Remarks**
SQUAL	0 × 04	0–254	Number of features in current frame
Maximum Pixel	0 × 05	0–63	Maximum pixel value in current frame
Minimum Pixel	0 × 06	0–63	Minimum pixel value in current frame
Pixel Sum	0 × 07	0–159	Full sum of pixel values/128 current frame
Shutter Upper	0 × 09	0–254	Read first upper 8 bits of 16 bit integration time
Shutter Lower	0 × 11	0–254	Read second lower 8 bits of 16 bit integration time
Image	0 × 08	0–63	Actual 361 pixel value array dump

**Table 2.** Flooring material test results, and room localization test results.

**Features Source**	**Percentage of Correctly Identified Samples**				**Number of Samples Tested**	

	***1-NN Classifier***	***Bayes-Normal-1***	***Bayes-Normal-2***	***Naive Bayes***	***Total***	***Average Per Floor Class***
**Initial floor covering test (52 classes) using the un-modified mouse sensor with new flooring materials in a controlled lighting environment**						

RGB and Registers	91.1	87.0	94.8	68.8	2,330	44
RGB	92.4	84.7	91.8	51.7	2,330	44
Mouse Registers	34.1	38.7	33.2	27.7	2,330	44

**Second floor covering test (50 classes) using the modified mouse sensor with new flooring materials in a controlled lighting environment**						

RGB and Registers	93.3	95.0	29.1	66.1	500	10
RGB	90.3	93.5	2.0	56.3	500	10
Mouse Image Texture	8.6	7.8	9.2	6.9	500	10
Mouse Registers	28.3	40.2	19.3	28.6	500	10
Webcam RGB + Texture	43.5	85.0	63.6	69.8	500	10
Webcam RGB	37.9	45.5	40.8	22.0	500	10
Webcam Texture	77.6	76.7	73.1	55.0	500	10

**Room localization testing (133 rooms and 35 classes of flooring), using the modified mouse sensor on flooring materials in various states of wear with un-controlled random background lighting**						

RGB and Registers	94.7	85.2	94.3	68.5	59,797	1,622
RGB	93.3	76.9	90.4	54.5	59,797	1,622
Registers	39.3	42.7	46.0	39.3	59,797	1,622

**Table 3.** Background lighting test results.

**Features Source**	**Percentage of Correctly Identified Samples**			

	***1-NN Classifier***	***Bayes-Normal-1***	***Bayes-Normal-2***	***Naive Bayes***
**Intense background daylight and localized LED illumination, no other light**				

RGB and Registers	94.1	98.2	90.9	82.7
RGB	90.5	99.5	97.7	78.2
Mouse Registers	35.9	51.8	29.1	39.1
Webcam RGB + Texture	93.6	91.8	85.0	88.2
Webcam RGB	93.6	94.1	93.2	80.0
Webcam Texture	64.1	62.3	81.4	62.3

**Intense background incandescent and LED illumination, no other light**				

RGB and Registers	81.8	88.2	78.2	76.8
RGB	78.6	80.5	45.9	65.0
Mouse Registers	27.3	26.4	33.2	32.3
Webcam RGB + Texture	78.6	97.7	85.9	94.1
Webcam RGB	79.5	80.0	79.5	66.8
Webcam Texture	65.5	70.9	68.2	62.3

**Typical office background fluorescent and LED illumination, no other light**				

RGB and Registers	90.3	78.0	87.1	55.9
RGB	85.6	58.8	83.8	42.6
Registers	30.0	32.1	37.9	29.1
Webcam RGB + Texture	84.1	95.0	85.5	90.0
Webcam RGB	85.0	87.3	80.9	75.5
Webcam Texture	88.6	94.5	90.5	78.6

**All lighting sources combined and LED illumination**				

RGB and Registers	94.7	85.2	94.3	68.5
RGB	93.3	76.9	90.4	54.5
Registers	39.3	42.7	46.0	39.3
Webcam RGB + Texture	84.1	82.0	92.0	86.2
Webcam RGB	82.3	54.9	74.5	58.9
Webcam Texture	66.8	38.5	63.2	51.4
